# Feasibility of Internet-Based Mind-Body Training for Adults With Subjective Cognitive Decline: Protocol for a Randomized Controlled Trial

**DOI:** 10.2196/86276

**Published:** 2026-04-13

**Authors:** Ruchika S Prakash, Madhura Phansikar, Megan E Fisher, Sarah Dove, Rebecca Andridge, Doug Scharre, Kathy D Wright, Elizabeth Head, Claire O'Callaghan, Quinn K Meyerson, Elizabeth J Duraney, Matthew Schroeder, Niyathi Chakrapani, Elise K Grant, Tyreek Jones, James Teng, Michael Esterman, Joshua M Smyth, Thomas Gregoire, Rond Malhas

**Affiliations:** 1 Department of Psychology, College of Arts & Sciences, The Ohio State University, 1835 Neil Avenue, Columbus, OH, 43210, United States, 1 6142928462; 2 Office of Curriculum and Scholarship, College of Medicine, The Ohio State University, Columbus, OH, United States; 3 Division of Biostatistics, College of Public Health, The Ohio State University, Columbus, OH, United States; 4 Department of Neurology, College of Medicine, The Ohio State University, Columbus, OH, United States; 5 College of Nursing, Wexner Medical Center, The Ohio State University, Columbus, OH, United States; 6 Department of Pathology and Laboratory Medicine, University of California, Irvine, Irvine, CA, United States; 7 School of Medical Sciences, Faculty of Medicine and Health, The University of Sydney, Sydney, Australia; 8 National Center for PTSD, VA Boston Healthcare System, Boston, MA, United States; 9 Department of Psychiatry, Boston University Chobanian & Avedisian School of Medicine, Boston, MA, United States; 10 College of Social Work, The Ohio State University, Columbus, OH, United States

**Keywords:** subjective cognitive decline, amyloid and tau pathology, online mind-body interventions, mindfulness, pilot randomized controlled trial

## Abstract

**Background:**

In the United States, the prevalence of Alzheimer disease (AD) is projected to double over the next 30 years, with associated familial and societal costs estimated at US $1 trillion annually if current trends continue. Although pharmacological treatments of AD are showing promise, the adoption of healthy lifestyle behaviors, particularly during the preclinical phase of AD, may reduce dementia rates by up to 45%. Subjective cognitive decline (SCD), defined as persistent self-perceived declines in cognitive functioning compared with previously normal cognitive abilities, has been identified as a potential preclinical stage of AD.

**Objective:**

This randomized controlled trial aims to evaluate the feasibility and acceptability of an internet-based, asynchronous mindfulness-based stress reduction program compared with an active control group (an internet-based lifestyle education program). Secondary objectives include examining preliminary effects of each intervention on SCD, plasma-based biomarkers of amyloid and tau pathology, and everyday mind-wandering.

**Methods:**

Sixty adults aged 50 years and older will be screened for SCD in the absence of objective cognitive impairment, based on the Uniform Data Set Neuropsychological Battery (version 3.0) from the National Alzheimer’s Coordinating Center. Eligible and consenting participants will complete behavioral and imaging-based tasks of sustained attention and mind-wandering, as well as blood draws at baseline and after the 8-week intervention. After baseline assessments, participants will be randomized to either an internet-based, asynchronous mindfulness-based stress reduction program or the internet-based lifestyle education program. Both programs have been adapted from our manualized in-person programs and refined through focus group interviews with the target population.

**Results:**

The study was funded in April 2024. Phase 1 focused on iterative development of the 2 programs based on focus group feedback. Recruitment for the randomized controlled trial (internet-based mind-body training trial) began in June 2025 and is ongoing. Recruitment is expected to conclude in September 2026, with data collection ending in December 2026.

**Conclusions:**

Behavioral, lifestyle-based interventions that emphasize experiential practices show promise as preventative strategies to prevent decline in cognitive and brain health. Yet, there remain significant barriers to engaging with in-person programs, including limited accessibility, time and schedule constraints, and travel logistics. The internet-based mind-body training trial will evaluate the feasibility and acceptability of 2 fully online, mind-body training programs for adults at risk for AD. Future Stage II and Stage III studies will be necessary to establish the efficacy of these programs for improving AD biomarkers and cognitive outcomes and their broader dissemination to adults noticing subtle changes in cognitive functioning.

## Introduction

**Figure 1.**
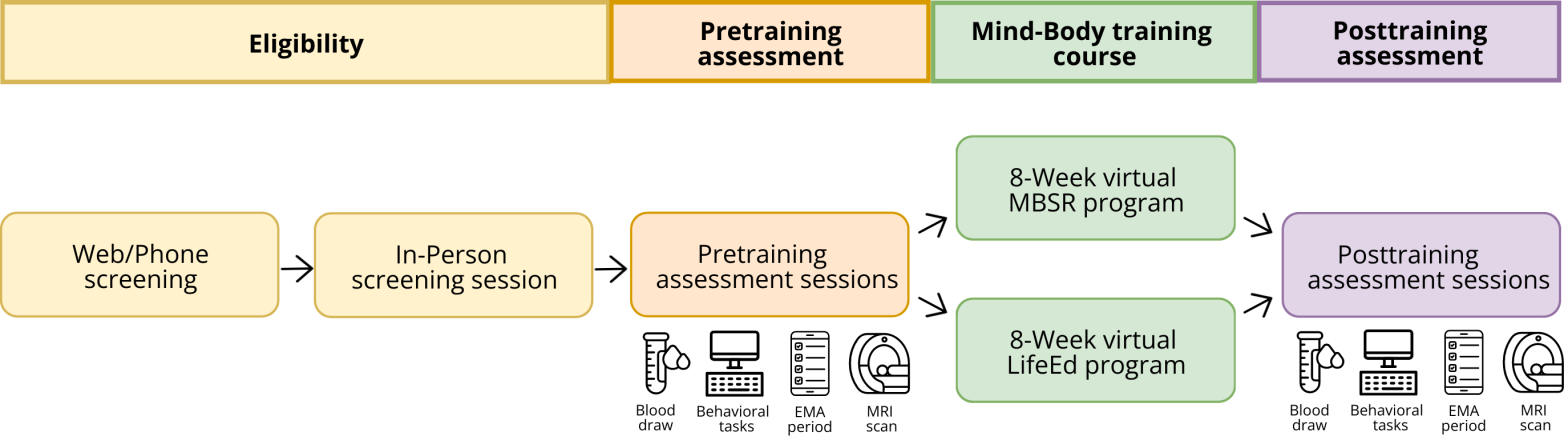
Timeline of the internet-based mind-body training randomized controlled trial, including screening sessions, baseline assessments, and intervention sessions. Following a combination of web or phone screening and an in-person screening session, participants complete behavioral and magnetic resonance imaging (MRI) sessions, which are repeated immediately after the 8-week training program. Blood and ecological momentary assessment (EMA) data are also collected before and after the intervention. LifeEd: lifestyle education; MBSR: mindfulness-based stress reduction.

Alzheimer disease (AD) is the leading cause of dementia worldwide, affecting 6.7 million Americans aged 65 years and older. According to the *2023 Alzheimer’s Disease Facts and Figures* [1], this number is projected to double by 2050, with individuals diagnosed with AD expected to comprise 22% of the US population. With limited treatment options, there has been a renewed focus on targeting neurodegenerative and pathophysiological processes through behavioral and lifestyle-based interventions during the prodromal phase of AD. These preclinical stages represent a window of opportunity for preventative interventions aimed at slowing the rate of accumulation of AD-related proteinopathies or delaying the functional impact of neurodegeneration on cognition [2].

Subjective cognitive decline (SCD), defined as perceived, persistent declines in cognitive functioning relative to one’s previous abilities, was first introduced in 2014 as a potential preclinical stage of AD [3]. Individuals with SCD are identified through self-reported concerns about cognitive functioning, typically assessed through administering simple paper-and-pencil questionnaires, such as the Everyday Cognition (ECog) Questionnaire [4], the Subjective Cognitive Decline Questionnaire [5], the Cognitive Change Index [6], or the Cognitive Function Index [7]. The classification of SCD additionally requires normal performance on standardized cognitive measures, a criterion that allows individuals with SCD to be distinguished from those with mild cognitive impairment (MCI) [3]. SCD has been associated later in life with the classic neural signatures of AD, including cortical thinning in key regions of the temporal lobe [8,9]; progressive whole-brain atrophy [10]; loss of gray matter volume, especially in the hippocampus [11]; and white matter hyperintensities [12]. It has also been associated with β-amyloid plaques [13,14] and other cerebrospinal fluid (CSF)–based AD biomarkers [15]. Moreover, it is estimated that 25% to 40% of adults seeking medical advice at Memory Disorders Clinics are reported to have SCD [16,17], which would allow for preventative interventions to be more easily implemented, potentially with higher rates of adherence compared to a community sample [2].

Mindfulness meditation, traditionally offered through manualized 8-week programs such as the mindfulness-based stress reduction program or the mindfulness-based cognitive therapy program, has a growing evidence base for its potential to benefit cognitive and affective health [18-20]. Mindfulness, as taught in these programs, involves the intentional cultivation of present moment awareness to fully observe and experience the unfolding of each moment [21]. Eight weeks of training in mindfulness practices typically include experiential exercises ranging from focused attention practices, which anchor attention to a single phenomenon (eg, the breath), to open monitoring practices, which involve flexibly switching attention to whichever phenomenon becomes most salient.

Accordingly, attention and its various components, including orientation, alertness, and executive control, as well as lapses in attention, often termed mind-wandering, have been investigated as primary outcome variables in many clinical trials of mindfulness meditation (see [22] for a scoping review). Results from randomized controlled trials (RCTs) in our laboratory have also demonstrated that mindfulness training reduces off-task thinking or mind-wandering [23,24], although in our Stage II efficacy study [24], these effects were not maintained at long-term follow-up. These reductions in mind-wandering propensity, replicated by other clinical trials (see Feruglio et al [25] for a systematic review), provide preliminary support that training in mindfulness meditation may strengthen goal maintenance systems, thereby enhancing one’s ability to prioritize task-relevant representations and filter out irrelevant, internal thoughts or external stimuli in the environment.

Additionally, across cross-sectional and intervention studies, mindfulness training has been shown to reduce activity within the default mode network (DMN) during tasks of sustained attention, strengthen intranetwork connectivity of the DMN, and increase connectivity between the DMN and the frontoparietal networks. In a cross-sectional study of healthy older adults, our laboratory provided the first evidence of trait mindfulness to be associated with greater intranetwork DMN connectivity [1,2,26]. Expert meditators with more than 10,000 hours of meditative practices consistently show reduced activity in the posterior cingulate cortex and the medial prefrontal cortex (core DMN hubs) both during cognitively demanding tasks [27] and while meditating in the magnetic resonance imaging (MRI) scanner [28]. Short-term RCTs in meditation novices have also shown reduced low-frequency amplitude within DMN regions [29] and increased negative connectivity between DMN and frontoparietal networks following the training [30,31].

There is also preliminary support for mindfulness training to strengthen the intranetwork connectivity of regions of the DMN, including the posterior cingulate cortex, the medial prefrontal cortex, and the hippocampus in individuals with MCI [32]. In the same study, 8 weeks of mindfulness training also resulted in the maintenance of hippocampal volume, a validated biomarker for clinical trials, including those conducted during predementia stages, compared to the volumetric decline observed in the control group. More recently, findings from the PREVENT-AD (Pre-symptomatic Evaluation of Experimental or Novel Treatments for AD) study established direct links between mindfulness and AD pathology in adults aged older than 60 years and at risk for dementia (ie, having either a parent or at least two siblings with AD-like dementia) [33]. Trait mindfulness, assessed using the Five Facet Mindfulness Questionnaire, was associated with a lower β-amyloid burden in the temporoparietal and frontal regions, including the precuneus, the posterior cingulate cortex, and the orbitofrontal cortex [34]. Facets of mindfulness were also associated with lower tau burden in the bilateral temporal regions, such as the entorhinal cortices; amygdala; and parahippocampal, inferior temporal, and fusiform gyri. Finally, trait mindfulness was also linked to reduced cognitive decline in global cognition, sustained attention, immediate memory, and delayed memory. This study thus provided the first direct evidence of a multivariate relationship among mindfulness, AD pathophysiology, and cognitive decline.

Building on these cross-sectional studies, our RCT, the internet-based mind-body training (iMBT) program, will evaluate the feasibility and acceptability of 2 fully asynchronous mind-body interventions: an internet-based mindfulness-based stress reduction (MBSR) program and an internet-based lifestyle education (iLifeEd) program. The 2 programs were co-developed with input from focus groups, including members of our target population, namely, adults aged 50 years and older with SCD. We will additionally examine preliminary effects on plasma Aβ_42_/Aβ_40_ and phosphorylated tau (p-tau217), biomarkers with high sensitivity and specificity in detecting AD amyloid and tau pathology, with validation studies showing a strong correspondence between plasma-based biomarkers and positron emission tomography (PET), CSF, and autopsy measures [35-37]. Other secondary outcomes include ecological momentary assessment (EMA) of mind-wandering and cognitive variability, DMN integrity during sustained attention, and behavioral measures of mind-wandering.

## Methods

### Study Design

The primary goal of our iMBT trial is to examine the feasibility and acceptability of the internet-based, asynchronous mindfulness-based stress reduction (iMBSR) program using a Stage I study design proposed in the National Institutes of Health (NIH) Stage Model [38]. Pilot studies are foundational for large-scale efficacy studies and help assess recruitment potential, delivery of the intervention, adherence and acceptability, barriers to compliance, and potential safety issues before the launch of large clinical trials [39-41]. These studies can also be used to generate estimates of a treatment’s effects and its variance and covariance to provide necessary data for large-scale trials [42]. For this RCT, we are recruiting a sample of adults with SCD, and participants are being randomized into 1 of 2 groups: iMBSR or iLifeEd. We are collecting data on the feasibility and acceptability of the program using a priori defined criteria. Additionally, blood plasma will also be collected before randomization and after 8 weeks of training to determine the effect of mindfulness training on Aβ_42_/Aβ_40_ ratio and p-tau217. To examine the preliminary effects of the iMBSR program on brain health, neuroimaging sessions will also be completed at both time points with the strength of the DMN serving as the measure of neuronal health. Participants will additionally complete computerized measures of sustained attention, EMA of mind-wandering and cognitive functioning, and several self-report questionnaires to assess their psychosocial profile. Figure 1 illustrates the timeline of assessment and intervention sessions for the ongoing clinical trial.

### Setting

All behavioral data are being collected in the Clinical Neuroscience Lab, located within the Department of Psychology at The Ohio State University. The Clinical Neuroscience Lab, directed by the study’s principal investigator (PI; RSP), contains 3 dedicated testing rooms equipped for neuropsychological testing. Blood samples are also obtained at the laboratory by trained phlebotomists from the Clinical Research Center at The Ohio State University. Neuroimaging data are being acquired at the Center for Cognitive and Behavioral Brain Imaging, an interdisciplinary research facility housed within the Department of Psychology at The Ohio State University.

### Participant Eligibility

The target population for this study is adults aged older than 50 years with SCD. Following the criteria identified in a study by Jessen et al [43] to recruit participants with SCD, we will administer the ECog Questionnaire to quantify self-reported deficits in cognitive functioning. The ECog Questionnaire is a self-reported and an informant-reported 39-item questionnaire assessing cognitively based everyday activities [41]. This measure was recently found to be reliable and valid in an ethnoracially diverse sample of older adults, with scores on this measure correlating reliably with concurrent and longitudinal changes in cognition and brain volumes [44]. In the proposed study, we chose to use the self-reported ECog Questionnaire to reduce participant burden. Emerging evidence suggests that inclusion criteria requiring input from informants, such as family members, disproportionately exclude traditionally underrepresented groups, including individuals with low socioeconomic status and those from racial and ethnic minority backgrounds [45,46]. Furthermore, for this study, we will use the previously validated cutoff score of 1.31 to recruit participants with SCD. This cutoff has been independently validated in 2 separate studies [47,48], with individuals scoring greater than 1.31 on the self-reported ECog twice as likely to be diagnosed with MCI [47]. Only participants with elevated scores (>1.31) will be invited for an assessment of objective cognitive performance.

To screen out potential cognitive impairment and keep participant burden to a minimum, participants who meet our cutoff for the ECog Questionnaire will first complete the Telephone Montreal Cognitive Assessment (T-MOCA) [49] and the Instrumental Activities of Daily Living (IADL) [50] to screen out individuals with potential AD and related dementias. The T-MOCA is a widely used screener that can be administered over the phone to assess potential cognitive impairment. Only participants who score greater than or equal to 19 on the T-MOCA (out of 22) and score 8 (out of 8) on the IADL are then invited for an in-person detailed neuropsychological assessment session where they are administered the Uniform Data Set Neuropsychological Battery (version 3.0; UDS3-NB) from the National Alzheimer’s Coordinating Center [51]. The UDS3-NB includes 10 widely used and well-standardized neuropsychological measures. Confirmatory factor analyses of the cognitive tests in the UDS3-NB in an ethnoracially diverse sample of more than 20,000 older adults from the National Alzheimer’s Coordinating Center identified a 4-factor latent structure, including the domains of attention, episodic memory, language, and processing speed/executive functioning [52]. Table 1 lists the specific measures and metrics that quantify performance in the 4 cognitive domains. Using the Jak/Bondi diagnostic criteria [53,54], participants will be classified as having MCI or dementia and excluded from the study if they score (1) higher than 1.5 SD below the demographically adjusted normative mean on all 3 measures of 1 domain or (2) higher than 1.5 SD below the demographically adjusted normative mean on at least one measure of all 4 domains.

**Table 1.** Cognitive domains, tasks, and specific metrics from the Uniform Data Set Neuropsychological Battery version 3.0 (National Alzheimer’s Coordinating Center) used to quantify objective cognitive performance.

Domain	Measure	Metric
Memory	Craft Story 21 Recall (immediate) Craft Story 21 Recall (delayed) Benson complex figure copy (delayed)	Verbatim score for the immediate recall Verbatim score for the delayed recall Total score for the delayed recall
Attention	Number span test: forward Number span test: backward Benson complex figure copy (immediate)	Number of correct trials Number of correct trials Total score for the Immediate Recall
Processing speed or executive functioning	Trail making test: part A Trail making test: part B Verbal fluency: phonemic test	Adjusted score: number of correct lines or times Adjusted score: number of correct lines or times Total number of correct “F” words and “L” words
Language	Multilingual naming test Category fluency Category fluency	Total score Total number of correct “animal” words Total number of correct “vegetable” words

### Sample Size and Power

Our feasibility study will involve 60 adults with SCD randomized to one of 2 groups: iMBSR or iLifeEd. Our primary outcome variables for this study are the feasibility and acceptability of the protocols. With 30 participants per group, we will be able to estimate within-group feasibility percentages (eg, the percentage of participants who find the program prospectively acceptable and the percentage of people who indicate adequate levels of satisfaction) with a margin of error of no larger than +18% or −18%. For intervention adherence, we will have 80% power for detecting a proportion of greater than 50% adherence if the true proportion of participants who complete all modules is 75%.

Our key secondary outcome variables for this study are the plasma Aβ_42_/Aβ_40_ ratio and plasma p-tau217, quantifying reduction in amyloid and tau pathology following training. We conducted sensitivity analyses to determine the effect size that we would need to observe to detect significant effects. Specifically, with 30 participants per group, we would need to observe a large effect size (Cohen *d*=0.74) to detect significant effects. Although there are, to our knowledge, no mindfulness-based interventions examining effects on plasma-based measures of amyloid and tau, prior work by one of the co-authors (EH) has demonstrated that 4 weeks of slow-paced breathing significantly decreases plasma Aβ_42^,^_ with a large effect size (Cohen *d*=0.81). As such, we are powered to detect the effect of our mindfulness intervention on plasma markers of amyloid. Other secondary variables of interest include behavioral and self-report measures of mind-wandering, including both laboratory- and EMA-based assessments of mind-wandering as well as the network strength of the DMN during the Gradual-Onset Continuous Performance Task. Given the pilot nature of the study, emphasis will be placed on the estimation of effect sizes and CIs for these effect sizes.

### Study Procedures

Participants who meet the demographic criteria, along with criteria for the ECog Questionnaire, the T-MOCA, the IADL questionnaire, and MRI safety, are invited to the Clinical Neuroscience Laboratory at The Ohio State University for an initial in-person screening session. At this visit, visual acuity is assessed using the Snellen chart, and a blinded research assistant administers the UDS3-NB.

Participants meeting criteria for intact objective cognitive performance are then invited to a behavioral testing session at the Clinical Neuroscience Lab and a neuroimaging session at the Center for Cognitive and Behavioral Brain Imaging. During the behavioral session, a trained nurse from the Clinical Research Center at The Ohio State University collects the blood plasma sample, after which participants complete 2 behavioral measures of sustained attention and mind-wandering. As psychoaffective factors can influence perceived ratings of SCD, participants also complete a battery of questionnaires assessing stress (the Perceived Stress Scale [55]), depression (the Center for Epidemiologic Studies Depression Scale [56]), anxiety (the Beck Anxiety Inventory [57]), worry (the Penn State Worry Questionnaire [58]), quality of life (the World Health Organization Quality of Life–Brief [59]), and perceived racial discrimination (the Everyday Discrimination Scale [60]). We also administer the Motivation to Change Lifestyle and Health Behaviors for Dementia Risk Reduction [61] to quantify psychological and cognitive factors that influence engaging in health behaviors.

At the same session, participants receive training on EMA procedures and are provided with study-specific smartphones (Samsung Galaxy phones) to collect EMA data on mind-wandering, cognitive functioning, perceived stress, affect, and arousal for 5 days between sessions. Finally, structural and functional MRI data are collected at the neuroimaging session to quantify changes in the integrity of the DMN.

After completion of all assessment sessions, participants are randomized to either the iMBSR or the iLifeEd group with a 1:1 allocation ratio. All assessments will be readministered immediately following the 8-week intervention.

### Randomization and Blinding

The study biostatistician (RA) generated the randomization sequence using a block size of 4 with stratification by sex. The project coordinator (EKG) received concealed envelopes for the 2 strata and is responsible for assigning eligible participants to one of the 2 groups. The project coordinator, the study facilitators, and the study biostatistician are the only team members unblinded to group assignment. All personnel conducting assessments, behavioral or neuroimaging, will remain blind to group assignment.

### Internet-Based Mind-Body Training Programs

For this study, we developed 2 asynchronous, 8-week online mind-body training programs: the iMBSR and the iLifeEd training programs. Both programs were provisionally adapted from our standardized MBSR and lifestyle education manuals, which have previously been used to conduct Stage II efficacy studies of mindfulness meditation [24,62]. Our team’s expertise in contemplative sciences, RCTs, and psychosocial interventions was complemented by instructional design expertise from the Office of Distance Education at The Ohio State University. In designing these platforms, we ensured inclusion of the critical components of the MBSR and lifestyle education program, including didactics, experiential learning, homework practices, social support and community building, and facilitator interactions. We additionally conducted focus groups with our target population, adults aged older than 50 years with SCD, to better understand their perspectives on healthy living, brain health, and engagement with digital interventions for improving brain health. Themes from the focus groups (N=23) were incorporated throughout the platforms, resulting in the final versions of the iMBSR and the iLifeEd programs.

The iMBSR and iLifeEd programs are delivered through The Ohio State University’s Canvas for Continuing Education platform. Each program consists of 8 modules, with each module requiring approximately 2 hours per week to complete. Participants are expected to complete 1 module per week over 8 weeks. In addition, they are invited to optional weekly drop-in hours with the study PI, a licensed psychologist with more than 17 years of experience facilitating mind-body interventions.

The 2 programs are matched on the following metrics: (1) format of delivery,—both programs are online and quasi–self-paced; (2) timing—each of the 8 modules for the 2 programs is expected to take 2 hours per week, plus optional 30-minute drop-in hours; (3) content—both programs have been designed to integrate didactics, experiential practices, and homework practices; and (4) peer support—participants in both groups have access to a community board for discussions following experiential practices. The community boards are expected to foster a sense of community throughout the program. Table 2 outlines the themes of each module for both programs and provides an overview of the content covered in each module.

**Table 2.** Weekly themes, practices, and homework assignments for each of the 8 weeks of the internet-based mindfulness-based stress reduction (iMBSR) and internet-based lifestyle education (iLifeEd) programs developed for the internet-based mind-body training study.

	iMBSR	iLifeEd
Module 1	The potential of mindfulness meditation Pt^a^ 1. Introduction to study aims (40 min) Pt 2. Defining mindfulness (45 min) Pt 3. Putting it into practice! (30 min) Hw^b^ 1. Mindful eating (15 min*)* Hw 2. Body scan practice (15 min*)*	Introduction to the study Pt 1. Introduction to study aims (45 min) Pt 2. Shift from the biomedical model to the biopsychosocial model (40 min) Pt 3. Watch, reflect, and apply! (40 min) Hw 1. Blue zones (20 min) Hw 2. Stretching and toning (15 min) Hw 3. On health literacy (5 min)
Module 2	Breath as our ever-present anchor Pt 1. Cultivating awareness by tuning into our bodies (35 min) Pt 2. Cultivating mindful awareness with the breath (50 min) Pt 3. Putting it into practice! (30 min) Hw 1. Body scan practice (15 min) Hw 2. Breath awareness practice (15 min)	All about sedentary behavior Pt 1. Introduction to sedentary behavior (40 min) Pt 2. Benefits of sitting less (30 min) Pt 3. Watch, reflect, and apply! (30 min) Hw 1. Posture for back pain (5 min) Hw 2. Stretching and toning (15 min) Hw 3. Move more, sit less (10 min)
Module 3	Mindful awareness of sensations Pt 1. Invitation to taking your seats (30 min) Pt 2. Cultivating awareness through mindful yoga (45 min) Pt 3. Putting it into practice! (35 min) Hw 1. Breath awareness practice (20 min) Hw 2. Mindful movement (15 min)	All about physical activity Pt 1. Introduction to physical activity (30 min) Pt 2. Benefits of physical activity and practical tips (35 min) Pt 3. Watch, reflect, and apply! (35 min) Hw 1. Exercising for brain health (5 min) Hw 2. Stretching and toning (15 min) Hw 3. Motivation for exercise (15 min)
Module 4	Mindful of emotions: impact on stress Pt 1. Understanding and experiencing emotions (35 min) Pt 2. Introduction to stress (45 min) Pt 3. Putting it into practice! (30 min) Hw 1. Mindful movement (15 min) Hw 2. Sitting meditation practice (15 min)	On brain plasticity Pt 1. Brain plasticity and cognitive reserve (40 min) Pt 2. Benefits of cognitively stimulating activities (30 min) Pt 3. Watch, reflect, and apply! (35 min) Hw 1. More on neuroplasticity (15 min) Hw 2. Stretching and toning (15 min) Hw 3. Interview with Yaakov Stern: founder of cognitive reserve (5 min)
Module 5	Finding the space to make choices Pt 1. Introduction to choiceless awareness (55 min) Pt 2. How do we contribute to our own stress? (25 min) Pt 3. Putting it into practice! (30 min) Hw 1. Choiceless awareness practice (15 min) Hw 2. Body scan practice (15 min)	All about sleep Pt 1. Introduction to sleep (40 min) Pt 2. Risks associated with sleeping less (35 min) Pt 3. Watch, reflect, and apply! (30 min) Hw 1. Busting sleep myths (5 min) Hw 2. Stretching and toning (15 min) Hw 3. One more reason to get good sleep (10 min)
Module 6	Firming and stabilizing your practice Pt 1. Deepening your meditation practices (45 min) Pt 2. More on breath awareness (40 min) Pt 3. Putting it into practice! (30 min) Hw 1. Standing movements (15 min) Hw 2. Choiceless awareness practice (15 min)	All about stress Pt 1. Introduction to stress (45 min) Pt 2. Coping with stress (25 min) Pt 3. Watch, reflect, and apply! (35 min) Hw 1. Why you need stress in your life (15 min) Hw 2. Stretching and toning (15 min) Hw 3. Mindfulness to reduce stress (5 min)
Module 7	Kindness toward self and others Pt 1. Starting with foundational practices (45 min) Pt 2. Practicing loving-kindness meditation (40 min) Pt 3. Putting it into practice! (30 min) Hw 1. Mindful yoga (15 min) Hw 2. Any 15-minute practice (15 min)	All about diet Pt 1. Introduction to diet – Focus on nutrition (45 min) Pt 2. Introduction to diet – Focus on hydration (35 min) Pt 3. Watch, reflect, and apply! (35 min) Hw 1. Why we can’t stop eating unhealthy foods (15 min) Hw 2. Stretching and toning (15 min) Hw 3. Dehydration with aging (5 min)
Module 8	Mindfulness: a lifelong practice! Pt 1. Returning to the basics (30 min) Pt 2. Final closing (50 min) Pt 3. Building your personalized mindfulness practice (30 min) Hw 1. Any 15-minute practice (15 min) Hw 2. Any 15-minute practice (15 min)	All about social support Pt 1. Introduction to social engagement and social support (30 min) Pt 2. Social engagement – Risks and practical strategies (20 min) Pt 3. Watch, reflect, and apply! (45 min) Hw 1. What makes a good life? (15 min) Hw 2. Stretching and toning (15 min) Hw 3. The value of human connection (15 min)

a Pt: part.

b Hw: homework.

### Primary Outcomes

Feasibility will be determined by evaluating the recruitment and retention of study participants, with high feasibility defined as the successful recruitment and enrollment of 60 participants and an attrition rate of no greater than 25%. We will also examine changes in SCD, with reductions on the ECog Questionnaire considered as evidence for feasibility of the intervention. Acceptability will be measured using the Acceptability of Intervention Measure [63], with high acceptability defined as an average score greater than 3 (on a 1‐5 scale, ranging from “completely agree” to “completely disagree”). Program satisfaction will be measured by our custom Postintervention Acceptability Questionnaire, which focuses on program enjoyment, satisfaction, and potential barriers. High program satisfaction will be defined as an average score greater than 5 (on a 0‐10 scale, ranging from “not at all” to “extremely” scale) for items focused on satisfaction. Participant attendance will also factor into acceptability, with high participation defined as at least 50% of participants successfully completing 6 or more modules on the class platform Scarlet Canvas.

### Secondary Outcomes: Plasma-Based Biomarkers of Amyloid and Tau

Blood draws are scheduled during the behavioral session. At each time point, we will collect 20 mL of blood (15 mL for plasma and 5 mL for serum). Following standard operating procedures, blood will be drawn via venipuncture from each participant into one 10 mL and one 5 mL lavender-top ethylenediaminetetraacetic acid tubes and one 5 mL red-top serum tube. Immediately after collecting blood, each ethylenediaminetetraacetic acid tube will be gently mixed by inverting 8 to 10 times to ensure proper mixing of blood and anticoagulant and kept at 4 °C. The red-top tube will also be gently mixed by inverting it 5 times and placed upright at room temperature for 30 minutes to allow for complete clot formation. Furthermore, within 24 hours of acquisition, all samples will be centrifuged for 10 minutes at 2000×*g* at 4 °C, and one-third of the aliquots will be shipped to our collaborators at the University of California, Irvine, for analysis. In this study, the secondary outcome variables are the plasma Aβ_42_/Aβ_40_ ratio, used to quantify amyloid pathology, and plasma p-tau217, used to determine tau pathology. Plasma samples will be analyzed using the automated Single Molecule Array (Simoa) HD-X analyzer with commercially available kits from Quanterix. A total of 100 µL of plasma will be used for the tau assays (p-tau217), and an additional 100 µL will be used for the 4-Plex panel.

### Other Outcomes

#### Behavioral and Imaging Measures of Sustained Attention and Mind-Wandering

##### Overview

Laboratory-based assessments of sustained attention and mind-wandering will include 2 behavioral tasks—the Shapes Expectation task [64,65], designed to place minimal demands on the participants, and the Go/No-Go task, a standardized task of sustained attention with embedded thought probes assessing mind-wandering. Participants will additionally complete the Gradual-Onset Continuous Performance Task in the MRI scanner.

##### Shapes Expectation Task

In this low-effort task, participants will simply be asked to focus on brightly colored geometric shapes on a computer screen. They will not be asked to remember the stimuli or respond to them. Following the presentation of the stimulus, a thought probe will appear on the screen instructing participants to report on the content of the thoughts they experienced while viewing the shape. This “thinking-aloud” technique in the context of a low-effort perceptual task has been shown to elicit a wide range of task-unrelated thoughts as well as to engage the activity of the 2 canonical networks critically implicated in mind-wandering—the default mode and the frontoparietal networks [64,65]. Participants will be provided with training examples before the commencement of the task. There will be a total of 9 trials with trials being of a short duration (≤20 s), a medium duration (30‐60 s), and a long duration (≥90 s). There will be no time limit imposed for the thought probes, and all responses will be recorded via a dictaphone.

##### Go/No-Go Task

In this task, participants will be presented with one of 2 visual stimuli (“X” and “M” or “Z” and “/”) for 750 ms, followed by an intertrial interval of 750 ms. They will be asked to press the corresponding key on frequent Go trials and to withhold responses on the less frequent (occurring 10% of the time) No-Go trials, which will be signaled by an auditory tone. Participants will complete 6 blocks of this task, with each block containing 54 Go trials, 6 No-Go trials, and 3 mind-wandering probes, resulting in a total of 18 mind-wandering probes. The quasi-randomly presented mind-wandering probes will ask participants to respond to categorize their immediately preceding thought as (1) “on task”; (2) “thinking about performance on the task”; or (3) “thinking about personal worries, daydreaming, fantasizing, or just lost in thought.”

##### Gradual-Onset Continuous Performance Task

In the MRI scanner, participants view a sequence of gradually appearing mountains and city scenes. They are asked to respond to frequently occurring city scenes (~90%) but to withhold their responses to mountain scenes [66,67]. Scenes transition gradually over 800 ms, and each of the 2 runs includes 9 blocks of varying durations (short: 44 s, medium: 52 s, and long: 60 s). At the end of each block, following the last trial, the scene transitions into a scrambled image, after which 2 thought probes are presented: (1) “To what degree was your focus just on the task or on something else?” and (2) “To what degree were you aware of your focus?” For the first probe, participants respond on a graded scale ranging from only else to only task, and for the second probe, from unaware to aware.

### Ambulatory Assessment of Mind-Wandering and Cognitive Variability

In alignment with recent recommendations for EMA-based studies [68], this study will collect EMA data 5 times per day over a 5-day period using study-provided smartphones. These assessments will be conducted before and after training. Participants will be provided with study-specific Android smartphones equipped with the MetricWire platform for EMA data collection, and they will undergo individualized training on EMA procedures. This training will help ensure participants can readily use the study application, understand the different measurements fully, and respond accurately to the EMA probes. The EMA platform will be programmed to signal participants with an audible beep and a pop-up notification at semirandom intervals throughout the day, structured into 5 time bins across waking hours. Mobile-based cognitive assessments of processing speed, working memory, and inhibitory control will include 3 tasks taken from the Mobile Monitoring of Cognitive Change (M2C2) battery [69]. The cognitive tasks will be completed immediately after each EMA report of mind-wandering.

### Mind-Wandering Prompts

#### Overview

Consistent with current best practice in EMA mind-wandering research [68,70,71], each signal will prompt participants to provide information about mind-wandering and contextual details from the moment they were signaled. At each signal, participants will be presented with the following probes: (1) In the moments just before the notification, to what degree had your mind wandered to something other than what you were doing? (2) In the moments just before the notification, to what degree were you actively directing what was on your mind? (3) In the moments just before the notification, to what degree were you aware of what was on your mind? and (4) In the moments just before the notification, to what degree were your thoughts moving freely? For each of these prompts, responses will be collected on a 5-point Likert-type scale, ranging from not at all (0) to very much so (4), with intermediate options of a little, somewhat, and quite a bit. Our primary variable will be the overall rate of mind-wandering in daily life.

#### Symbol Search Task

This is a speeded performance task, measuring processing speed and attention. Participants will be presented with a set of 3 symbol pairs located on the upper half of the screen and 2 symbol pairs on the lower half of the screen. They are instructed to select which of the bottom symbol pairs matches 1 of the 3 symbol pairs above, as quickly and accurately as possible. A total of 20 trials with an intertrial interval of 200 ms will be administered, and each set of stimuli will be presented until the participant selects a response. The SD of response times will be computed, with higher scores reflecting higher processing speed variability.

#### Grid Memory Task

This task is a delayed short-term recall task and measures visual working memory abilities. First, participants complete a 3-second encoding phase in which they are presented with a 5×5 grid containing 3 red dots. Participants are instructed to remember the locations of the 3 red dots. Second, participants complete an 8-second distraction phase where they are presented with a 5×6 grid of “F” letters among an array of “E” letters and are directed to select all the “F” letters as quickly as possible. Finally, they complete a retrieval phase where they are presented with an empty 5×5 grid and must indicate the location of the 3 red dots; this grid will be displayed until the participant submits their selection. A total of 4 trials will be administered. The SD of the Euclidean distance of the location of the incorrect dot to the correct dot location will be computed, with higher scores indicating more variable placement.

#### Letter Go/No-Go Task

This task is a continuous performance task measuring attention and inhibitory control. Participants will be presented with a series of 30 letters, one at a time, and are instructed to tap a button as quickly as possible when they are presented with each letter. However, when the participant is presented with the letter “X,” they are instructed to withhold their response. Each letter will be presented for 1000 ms with an intertrial interval of 500 ms, and the ratio of Go/No-Go trials is 5:1.

### Data Management and Analysis

Our laboratory is dedicated to conducting rigorous and reproducible randomized clinical trials that evaluate the feasibility and efficacy of lifestyle-based psychosocial interventions. We have established detailed procedures for data collection, reduction, storage, and analysis, and these protocols will be followed in this study.

All data collected as part of this study, including behavioral, self-report, and neuroimaging data, will be stored in the PI’s laboratory, accessible only to authorized laboratory personnel. Blood samples will be stored at the Clinical Research Center, and deidentified EMA data will be housed on MetricWire’s secure cloud server. For screening and behavioral assessments, 2 independent raters will code the measures (either manually or via custom scripts), with a third rater confirming agreement. Questionnaire data will be coded using custom scripts that have been independently reviewed by 2 laboratory members and validated against manual coding. EMA data will be processed using tools provided by MetricWire and subsequently analyzed with custom scripts.

Structural MRI and functional MRI (fMRI) preprocessing will be performed using the standardized fMRIPrep (version 1.4.1) pipeline [72]. T1-weighted (T1w) images will be corrected for intensity nonuniformity [73], skull-stripped (with brain surfaces reconstructed using FreeSurfer, version 6.0.1) [74], and spatially normalized to the Montreal Neurological Institute (MNI) space through nonlinear registration [75]. Functional data will be corrected for susceptibility distortion [76] and motion [77] and co-registered to the T1w using boundary-based registration [78]. Motion-correcting transformations, BOLD-to-T1w transformations, and T1w-to-template (MNI) warps will be concatenated and applied in a single step using Lanczos interpolation (ANTs, version 2.1.0). Physiological regressors [79], framewise displacement [80], and global signal will be extracted from the fMRI data. Preprocessed fMRI data will then be denoised using the signal clean function within Nilearn [81]. Potentially confounding sources of signal variance will be removed: mean CSF, white matter, global signal, a 24-parameter motion model, and the linear trend. To further control for motion-related confounds, we will add a spike regressor for each frame with a framewise displacement of ≥0.5 mm [80]. A high-pass temporal filtering at 0.01 Hz will also be applied.

Preprocessed fMRI data will then be parcellated using the Glasser functional atlas with 360 cortical regions and 19 subcortical regions [82,83]. The functional atlas will be transformed from MNI space to each participant’s native space. We will then extract the BOLD signal time courses from each of the 379 regions by averaging the signal across all voxels within each region. To compute the statistical dependence between BOLD signal across 2 nodes, we will first z-score each time series, compute their element-wise product, and then average this product to obtain the Pearson correlation coefficient between each pair of nodes. Calculating the Pearson correlation for all pairs of nodes will result in a 379×379 functional connectivity matrix for each participant, which will be used to compute the DMN connectivity.

Following the preprocessing of self-report, behavioral, and neuroimaging data, analyses will be conducted to determine intervention feasibility and acceptability. For preliminary effects on plasma biomarkers of AD pathology, behavioral and neural metrics of mind-wandering, and other secondary outcomes, we will fit linear mixed models to account for within-subject correlations arising from measuring each subject at 2 time points. In these models, emphasis will be placed on estimating effect sizes and CIs, rather than on hypothesis testing. As we are including adults with SCD based on self-reported concerns, we will also conduct sensitivity analyses using the total score on the Penn State Worry Questionnaire [58] to examine whether feasibility, acceptability, and preliminary effects differ as a function of worry scores within the current sample.

### Adverse Event Reporting

Adverse events (AEs) during the clinical trial will be reported in accordance with a detailed protocol developed in alignment with the NIH Adverse Event Reporting Guidelines. Any event brought to the study team’s attention will be immediately reported to the study PI and the clinical trial safety monitor. The safety monitor, a cognitive neurologist with extensive expertise in neurocognitive disorders, will evaluate the event and determine its relationship to the study. Each event will be classified as unrelated, possibly related, probably related, or definitely related using standard criteria for clinical trials.

For all events deemed possibly, probably, or definitely related to the study, they will then be categorized by severity as (1) mild AE—does not require treatment; (2) moderate AE—resolved with treatment; (3) severe AE—inability to carry on normal activities and requires professional medical attention; (4) life-threatening or disabling AE; and (5) fatal AE. For mild AEs, we will document the event, and the study team will determine whether changes to the study procedures are warranted. For moderate or more severe events that are related to the study, a detailed report will be prepared and submitted to the institutional review board (IRB) and the NIH Program Officer assigned to the study. If 2 participants experience the same AE, recruitment will be stopped, a report will be submitted to the IRB and NIH, and any revisions to the study protocol suggested by either the IRB or the NIH will be implemented. In addition, annual reports to the IRB and progress reports to the NIH will be submitted, including information on enrollments, withdrawals, completions, and the occurrence of AE.

### Ethical Considerations

The Ohio State Institutional Review Board has granted approval to conduct this study (IRB 2024H0401). All participants will provide written informed consent that details the aims of the study, outlines the elements of the study protocol, and describes the risks and benefits associated with participation. Participants are informed both verbally and through the written consent document that participation is voluntary in nature and that they may withdraw from the study at any time without penalty. All study personnel are trained in the ethical conduct of research and are explicitly prohibited from using any kind of coercion during study participation. Participants receive compensation at a rate of US $15 per hour for the in-person screening session, behavioral sessions, and MRI sessions (both before and after assessment). For EMA, participants receive US $10 per day for completing at least 4 of the 5 daily prompts (up to US $50 before and after assessment). Thus, for completing all pre- and post-assessments, participants can receive up to US $310. Enrolled participants can also receive additional compensation for referring others to participate in the study (US $5 per referral, up to US $35). Intervention sessions are provided to participants at no cost.

Although identifying information is collected for the study, where possible, data are collected using coded IDs. All data are stored on a secure cloud server housed at The Ohio State University. Upon study completion, all data will be fully deidentified using the Safe Harbor approach, and only coded data will be retained for study analyses.

## Results

This study was funded by the National Institute on Aging in April 2024. Phase I focused on iterative development of the 2 programs based on focus group feedback. Recruitment for the Phase II RCT (iMBT) began on June 13, 2025, and is ongoing. As of December 1, 2025, we have 31 participants randomized into the study: 15 participants have been randomized to the iMBSR group, and 16 participants have been randomized to the iLifeEd group. We anticipate that recruitment will end in September 2026, with all 60 participants randomized by that date. Although data analyses will begin after all participants have completed the intervention and assessment sessions, all analysis scripts have been finalized, and data reduction and processing are ongoing. We expect to complete the processing and analysis of the primary outcome variables by March 2027, with a targeted manuscript submission date of June 2027.

## Discussion

AD is a growing public health concern, particularly as targeted pharmacological interventions have not demonstrated their expected effects. Preventative interventions, targeting at-risk individuals when pathological processes are only beginning to manifest, have the potential to stave off the functional impairments associated with AD neurodegeneration. In this Stage I RCT, iMBT, we aim to systematically test the feasibility, acceptability, and preliminary effects of an internet-based mindfulness program. Sixty adults with SCD, a well-established late preclinical phase of AD, will be recruited. Participants will asynchronously complete 8 modules of either the iMBSR or the iLifeEd program, with behavioral and neuroimaging assessments conducted before and immediately after the intervention. Our main hypothesis is that iMBSR, carefully curated to include the active components of the MBSR program and designed to be self-paced and online, will be feasible and acceptable to adults at higher risk for developing AD and related dementias.

MBSR is a manualized 8-week program that provides systematic and incremental training in the principles, attitudinal foundations, and practices of mindfulness meditation [84,85]. These programs are traditionally taught in group-based settings with weekly 2.5-hour sessions. Despite its demonstrated clinical utility, the in-person format poses significant accessibility challenges, resulting in a growing need for more widely available adaptations [86]. Internet-based mindfulness programs offer a promising and cost-effective solution and have been rated as a preferred delivery format among the general population [86]. Several internet-based interventions, ideographically tailored for the target population, including acquired brain injury [87], fibromyalgia [88], and heart disease [89], show promising support. However, key methodological limitations necessitate the systematic testing of the iMBSR and iLifeEd protocols developed for this trial.

First, much of the extant literature on web-based mindfulness interventions lacks active control conditions, thus failing to account for nonspecific factors and expectancy effects [22]. For example, in one of the largest internet-delivered mindfulness trials, 150 individuals with cancer reported reductions in self-reported anxiety and depression [90]. However, because comparisons were made against a wait-list control group, it is unclear whether improvements reflected mindfulness practices, demand characteristics, or even placebo effects. Second, the majority of existing internet-based mindfulness training programs are based on mindfulness-based cognitive therapy [91-93] and acceptance and commitment therapy [94-96], or they simply teach participants how to meditate [97,98]. In contrast, surprisingly few studies have adapted MBSR for internet delivery.

Of the existing internet MBSR adaptations, most are synchronous formats [87,99,100], whereas only one study, to our knowledge, has developed an asynchronous internet-based MBSR program [101]. In that study, participants completed 30 minutes of class material per week compared to the 2.5 hours common among standard in-person programs, thus questioning the treatment fidelity of this program [102]. Although synchronous formats more closely resemble traditional in-person mindfulness-based programs in terms of course content and class time, they fail to offer participants the flexibility to complete components on their personal schedule. Asynchronous formats may be particularly advantageous for individuals unable to commit to predetermined group meeting times, as is common among midlife adults (a sample included in this RCT). Finally, only approximately half of internet-based interventions report on participant adherence [103], limiting conclusions about their efficacy in targeting key outcomes. In fact, in a systematic review of all psychosocial wellness and stress management applications, only 2.08% (21 of 1009) mobile apps had published, peer-reviewed evidence of feasibility and/or efficacy [104]. There is thus a critical need to develop and rigorously test an internet-based mindfulness program that is derived from the standardized 8-week MBSR protocol, designed for the target population (here, adults aged ≥50 years with SCD), including an active control comparison matched for nonspecific factors, and has in-built elements for increasing and maintaining adherence. Leveraging our team’s expertise in delivering in-person mindfulness programs and designing mobile and web-based apps for the delivery of health-based interventions [24,105,106], the iMBT trial incorporates state-of-the-art research elements into an internet-delivered, guided, self-paced mindfulness program for adults at risk for AD. We have also designed a comparable control program, created by the same team and focus group participants, against which we will compare the iMBSR program in future studies to ensure internal validity before making causal claims about efficacy.

In addition to examining feasibility and acceptability, this study will examine the preliminary effects of mindfulness practice on amyloid and tau pathology. The deposition of these proteins in the densely connected nodes of the DMN is a characteristic hallmark of AD [107,108]. Early aggregation of amyloid has been observed in anterior midcortical structures, such as the medial orbitofrontal gyrus and the anterior cingulate cortex, as well as in posterior midcortical structures, including the posterior cingulate cortex and the precuneus. Tau pathology is known to spread in a prion-like manner, beginning in the transentorhinal cortex and progressing to the perirhinal cortex, hippocampus, limbic areas, and, eventually, the association cortices [109-112]. The spatial affinities of Aβ and tau-based tangles for DMN regions, which also appear to support the prophylactic benefits of mindfulness meditation [26-31], provide the scientific rationale for this RCT. We hypothesize that mindfulness training will facilitate present moment awareness and reduce mind-wandering in daily life, thereby strengthening DMN connectivity and exerting downstream cascading effects on amyloid and tau pathology.

AD pathology in this study will be quantified using the recently validated plasma-based biomarkers of Aβ and tau [113-117]. Emerging evidence demonstrates that low plasma Aβ (Aβ_42_/Aβ_40_) levels are associated with higher brain amyloid deposition as measured using PET imaging [118-120]. Although high levels of p-tau217 show a positive association with amyloid PET data, growing evidence also suggests that plasma p-tau217 is even more closely associated with brain tau deposition, particularly in later disease stages [115,121,122]. Importantly, these plasma-based measures were recently validated in a multiethnic community study of 300 participants that included 46% non-Hispanic White participants and 54% participants from other ethnoracial groups [123]. The iMBT clinical trial will provide the first test of whether online mindfulness training can reduce amyloid and tau pathology in adults at risk for AD.

In conclusion, the iMBT trial is designed to test the feasibility and acceptability of 2 asynchronous, online mind-body training programs. Both interventions were systematically adapted from in-person formats with demonstrated high adherence rates [62] and further refined for online delivery using instructional design principles and feedback from the target population. Findings from this trial will provide critical groundwork for disseminating these accessible interventions to adults reporting everyday cognitive concerns. Given the limited availability of evidence-based preventative programs, these interventions have the potential to expand access to scalable, mind-body interventions for adults at risk for cognitive impairment.

## Abbreviations

AD: Alzheimer disease
AE: adverse event
CSF: cerebrospinal fluid
DMN: default mode network
ECog: Everyday Cognition
EMA: ecological momentary assessment
IADL: Instrumental Activities of Daily Living
IRB: institutional review board
MBSR: mindfulness-based stress reduction
MCI: mild cognitive impairment
MNI: Montreal Neurological Institute
MRI: magnetic resonance imaging
NIH: National Institutes of Health
PET: positron emission tomography
PI: principal investigator
RCT: randomized controlled trial
SCD: subjective cognitive decline
T-MOCA: Telephone Montreal Cognitive Assessment
T1w: T1-weighted
UDS-NB: Uniform Data Set Neuropsychological Battery
